# Potential drivers of vector-borne pathogens in urban environments: European hedgehogs (*Erinaceus europaeus*) in the spotlight

**DOI:** 10.1016/j.onehlt.2024.100764

**Published:** 2024-05-23

**Authors:** Andrea Springer, Karolin Schütte, Florian Brandes, Maximilian Reuschel, Michael Fehr, Gerhard Dobler, Gabriele Margos, Volker Fingerle, Hein Sprong, Christina Strube

**Affiliations:** a Institute for Parasitology, Centre for Infection Medicine, University of Veterinary Medicine Hannover, Buenteweg 17, 30559 Hanover, Germany; b Wildlife Rescue and Conservation Center Sachsenhagen, Hohe Warte 1, 31553 Sachsenhagen, Germany; c Department of Small Mammal, Reptile and Avian Diseases, University of Veterinary Medicine Hanover, Buenteweg 9, 30559 Hanover, Germany; d National Reference Laboratory for TBEV, Bundeswehr Institute of Microbiology, Neuherbergstr. 11, 80937 Munich, Germany; e National Reference Center for *Borrelia*, Bavarian Food and Health and Food Safety Authority, Veterinärstraße 2, 85764 Oberschleissheim, Germany; f Centre for Infectious Disease Control, National Institute of Public Health and Environment, Antonie van Leeuwenhoeklaan 9, 3720, BA, Bilthoven, Netherlands

**Keywords:** Tick-borne pathogens, Zoonoses, *Borrelia*, *Anaplasma phagocytophilum*, *Rickettsia*, *Bartonella*

## Abstract

Vector-borne diseases (VBDs) are considered as (re-)emerging, but information on the transmission cycles and wildlife reservoirs is often incomplete, particularly with regard to urban areas. The present study investigated blood samples from European hedgehogs (*Erinaceus europaeus*) presented at wildlife rehabilitation centres in the region of Hanover. Past exposure to *B. burgdorferi* sensu lato (s.l.) and tick-borne encephalitis virus (TBEV) was assessed by serological detection of antibodies, while current infections with *Borrelia* spp., *Anaplasma phagocytophilum*, *Rickettsia* spp., *Neoehrlichia mikurensis*, *Bartonella* spp., *Babesia* spp. and *Spiroplasma ixodetis* were investigated by (q)PCR. Of 539 hedgehogs tested for anti-*Borrelia* antibodies, 84.8% (457/539) were seropositive, with a higher seropositivity rate in adult than subadult animals, while anti-TBEV antibodies were detected in one animal only (0.2%; 1/526). By qPCR, 31.2% (168/539) of hedgehog blood samples were positive for *Borrelia* spp., 49.7% (261/525) for *A. phagocytophilum*, 13.0% (68/525) for *Bartonella* spp., 8.2% for *S. ixodetis* (43/525), 8.0% (42/525) for *Rickettsia* spp. and 1.3% (7/525) for *Babesia* spp., while *N. mikurensis* was not detected. While further differentiation of *Borrelia* spp. infections was not successful, 63.2% of the *A. phagocytophilum* infections were assigned to the zoonotic ecotype I and among *Rickettsia* spp. infections, 50.0% to *R. helvetica* by ecotype- or species-specific qPCR, respectively. Sequencing revealed the presence of a *Rickettsia* sp. closely related to *Rickettsia felis* in addition to a *Bartonella* sp. previously described from hedgehogs, as well as *Babesia microti* and *Babesia venatorum*. These findings show that hedgehogs from rehabilitation centres are valuable sources to identify One Health pathogens in urban areas. The hedgehogs are not only exposed to pathogens from fleas and ticks in urban areas, but they also act as potent amplifiers for these vectors and their pathogens, relevant for citizens and their pets.

## Highlights

• Hedgehogs in urban areas are potent reservoirs of One Health pathogens
• 84.8% of hedgehogs had anti-*Borrelia* antibodies, 31.2% were qPCR-positive
• 49.7% were *Anaplasma phagocytophilum* qPCR-positive
• *Rickettsia*, *Bartonella*, *Spiroplasma* and *Babesia* spp. were also detected
• Prevention of infected vector transfer to citizens and their pets is of One Health relevance

## Graphical abstract

**Figure:**
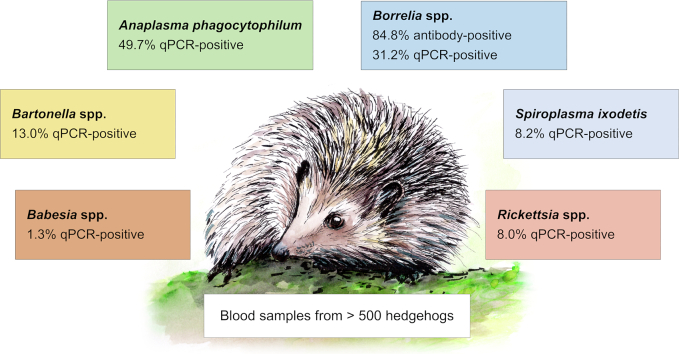

## 1 Introduction

Urbanization creates complex challenges for human, animal and environmental health including heat stress, increased pollution, habitat fragmentation and altered host communities for pathogens [[1], [2], [3]]. Within cities, urban green spaces increase human wellbeing by improving air and water quality, regulating the climate and fostering physical and mental health [4], but they also increase urban biodiversity by providing habitats for wild vertebrates and invertebrates, which may entail the risk of zoonotic disease transmission [5,6]. Vector-borne diseases (VBDs) are considered as (re-)emerging and of increasing One Health importance in Europe, affecting both human and animal health and involving invertebrate vectors as well as wildlife reservoir hosts [[7], [8], [9], [10]]. Lyme borreliosis (LB), caused by spirochetes of the *Borrelia burgdorferi* sensu lato (s.l.) complex and primarily transmitted by the widespread hard tick *Ixodes ricinus*, is the most frequent VBD on the continent, with an estimated 24% of the European population living in areas of high LB incidence [11]. Data from different countries show that a substantial share of *Borrelia* infections leading to LB cases is acquired in urban or peri-urban areas [12,13]. The annual economic burden resulting from LB-associated healthcare and indirect costs has been estimated at 0.14–1.36 USD per capita in different European countries [14]. In addition, a variety of other pathogens relevant for human and/or veterinary health circulates between *I. ricinus* and vertebrate reservoir hosts, including the relapsing-fever spirochete *Borrelia miyamotoi*, bacteria of the order Rickettsiales (*Anaplasma phagocytophilum*, *Rickettsia helvetica*, *Rickettsia monacensis*, *Neoehrlichia mikurensis*), tick-borne encephalitis virus (TBEV) and various *Babesia* species [15]. Recently, the tick endosymbiont *Spiroplasma ixodetis* has also been implicated in human disease cases [16].

Often, the natural transmission cycles and involved reservoir hosts of these pathogens are not fully known [17], particularly in urban and peri-urban areas which are characterized by a high human and pet density, but often a different and less diverse wildlife community composition compared to rural areas [18]. The European hedgehog (*Erinaceus europaeus*) is omnipresent in urban and peri-urban habitats, where its population density is often even higher than in rural landscapes [19,20]. Their preference for residential gardens and city parks results in a strong overlap with the environment of humans and their pets [21] and they are often taken into human care if found weak or injured [[22], [23], [24]]. Hedgehogs show a high ectoparasite prevalence and often a high infestation intensity [24,25]. In contrast to smaller mammals like rodents, which usually act as hosts for immature *I. ricinus* only [26], hedgehogs sustain all life stages of *I. ricinus* and of the hedgehog tick *Ixodes hexagonus* [24,25], thereby acting as a potential source of ticks in their local environment. Like *I. ricinus*, *I. hexagonus* is a vector of *B. burgdorferi* s.l. [27,28]. Due to its specialized, nest-adapted lifestyle, humans are only occasionally bitten by *I. hexagonus* [29,30], however, this species represented 5.5% and 1.6% of ticks collected from domestic cats and dogs in Germany, respectively [31].

The reservoir function of hedgehogs for *B. burgdorferi* s.l. has been proven by transmission to xenodiagnostic *I. hexagonus* as well as *I. ricinus* larvae [32]. Moreover, previous studies investigating European as well as Northern white-breasted hedgehogs (*Erinaceus roumanicus*) revealed high infection rates with *A. phagocytophilum* [[33], [34], [35]], indicating that hedgehogs potentially play an important epidemiological role for this pathogen. This is further corroborated by a higher *A. phagocytophilum* prevalence in engorged *I. ricinus* collected from European hedgehogs compared to questing ticks [25]. Similarly, DNA of *R. helvetica*, and certain members of the *B. burgdorferi* s.l. complex (*B. afzelii*, *B. bavariensis* and *B. spielmanii*) was also detected at higher prevalence in engorged ticks from hedgehogs than in questing ticks [25], although presence of DNA in engorged ticks does not necessarily indicate reservoir competence.

In addition, several *Bartonella* spp., which are primarily transmitted by fleas, have been detected in *E. europaeus* and *E. roumanicus*, including species with validated zoonotic potential [36]. Further *Bartonella* isolates from hedgehogs may also be of zoonotic relevance, as the number of *Bartonella* spp. associated with human disease continues to grow [37]. When hedgehogs are taken into human care [[22], [23], [24]], they may represent a source of *Bartonella* infection for humans via transfer of fleas or via bites and scratches.

The objective of the present study was to shed further light on the reservoir role of European hedgehogs by determining past and present infections with vector-borne pathogens as well as seasonal and age-related patterns in infection rates. Therefore, the present study investigated blood samples from >500 European hedgehogs presented at wildlife rehabilitation centres in or near the city of Hanover, Northern Germany, during 2018–2021. Past exposure to *B. burgdorferi* s.l. and TBEV was assessed by serological detection of antibodies, while molecular methods were employed to assess current infections with *Borrelia* spp., *A. phagocytophilum*, *Rickettsia* spp., *N. mikurensis*, *Bartonella* spp., *Babesia* spp. and *S. ixodetis*. The findings help to assess whether hedgehogs are a valuable tool to monitor the presence of One Health pathogens in urbanized areas. If so, then the potential contribution of hedgehogs to the One Health risk of these pathogens in urban areas can be assessed and taken into account by municipalities, citizens, veterinarians, and medical doctors.

## 2 Material and methods

### 2.1 Hedgehog sampling

Hedgehogs were examined at three wildlife rehabilitation centres located in or near the city of Hanover, capital of the federal state of Lower Saxony, Germany, between July 2018 and May 2021 [24]. Hedgehogs were brought into these rehabilitation centres due to weakness, illness or injury and subjected to a clinical examination and appropriate treatment. Based on a combination of body weight, body condition, teeth condition and season, hedgehogs were classified as juvenile, subadult or adult as described by Schütte et al. [24].

**Fig. 1:**
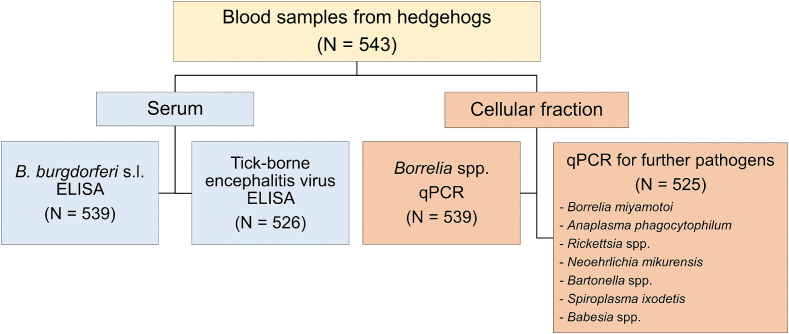
Overview of the study design investigating vector-borne pathogens in European hedgehogs from Northern Germany.

During the clinical examination, up to 2 ml of blood were taken from the saphenous vein or, in case the animal had to be euthanized or died prior to the examination, by puncture of the heart. Blood sampling was conducted in accordance with the German Animal Welfare act as well as the national and international guidelines for animal welfare and was approved by the ethics commission of the Animal Care and Use Committee of the Lower Saxony State Office for Consumer Protection and Food Safety (*Niedersächsisches Landesamt für Verbraucherschutz und Lebensmittelsicherheit*) under reference number 33.8‐42502-05-18A320. The blood samples were centrifuged at 2000 x*g* for 5 min. Serum and blood clots were stored separately at −20 °C for antibody ELISA and PCR investigations, respectively (Fig. 1).

### 2.2 Serological assays

The VetLine *Borrelia* ELISA kit (NovaTec Immundiagnostica GmbH, Dietzenbach, Germany) was used to detect IgG antibodies against *B. burgdorferi* s.l. according to the manufacturer's instructions, with test results interpreted as follows: > 11 NovaTec units (NTU) were considered positive, 9–11 NTU as borderline and < 9 NTU as negative.

For detection of anti-TBEV antibodies, the Immunozym FSME IgG All Species ELISA kit (Progen, Heidelberg, Germany) was used. Test interpretation followed the manufacturer's recommendations, with <63 Vienna units (VIEU)/ml considered negative, 63–126 VIEU/ml as borderline and > 126 VIEU/ml as positive. Samples with ≥60 VIEU/ml were subjected to serum neutralization test (SNT) at the National Reference Laboratory for TBEV to exclude false-positive results due to cross-reactions with antibodies against other flaviviruses. The SNT was conducted as a micro-neutralization test in 96-well plates as previously described [38]. Briefly, sera were inactivated at 56 °C for 30 min, followed by 1:10 and 1:20 dilution using cell culture medium (MEM supplemented with antibiotics, antimycotics and non-essential amino acids; Invitrogen, ThermoFisher Scientific, Darmstadt, Germany). Serum dilutions were incubated in duplicate for 1 h at 37 °C together with 50–100 tissue culture infectious doses of TBEV strain Neudörfl. After addition of 10^4^ A549 cells/well, the serum-virus solution was incubated for 5 days at 37 °C. Cells were fixed and stained using a 13% formalin-0.1% crystal violet solution, resulting in a prominent blue well for antibody-positive sera, while antibody-negative sera remained transparent. A positive result at 1:20 dilution was considered anti-TBEV antibody-positive. Sera with a titre of 1:10 were repeated in triplicate to confirm this titre and were then also classified anti-TBEV antibody-positive. Sera with titres <1:10 were classified as anti-TBEV antibody-negative.

### 2.3 DNA extraction and qPCR for vector-borne pathogens

DNA was extracted from blood clots, whereby the weight of each blood clot used for DNA extraction was determined and documented. The NucleoSpin Blood kit (Macherey-Nagel, Düren, Germany) was used according to the manufacturer's instructions.

Conducted quantitative real-time PCR (qPCR) analyses for screening of the samples are summarized in Table 1. For detection of *Borrelia* spp., the 23S rDNA and intergenic spacer (IGS) region was targeted by probe-based qPCR using 10 μl template as described previously [[39], [40], [41]]. Plasmid standards with 10^0^ to 10^6^ copies were included for quantification. Additionally, a *B. miyamotoi*-specific qPCR [42] was conducted for a subset of 525 samples. These 525 samples were additionally tested for *A. phagocytophilum*, *Rickettsia* spp., *N. mikurensis*, *S. ixodetis* and *Babesia* spp. by probe-based qPCR. For *A. phagocytophilum*, the *msp2* gene was targeted [43]. *Anaplasma*-positive samples were subsequently subjected to a qPCR protocol distinguishing between ecotype I and II by use of two different probes [44]. Regarding *Rickettsia* spp., two different qPCRs based on the *gltA* gene were employed [45,46], while the *groEL* gene was targeted for *N. mikurensis* [47], the *ssrA* gene for *Bartonella* spp. [48] and the *rpoB* gene for *Spiroplasma* spp. [49]. For detection of *Babesia* spp., the *18S* gene was amplified [50] and the *ITS* for *B. microti* [51].

**Table 1:** Detection and differentiation methods used for different vector-borne pathogens in blood samples of European hedgehogs.

Pathogen	qPCR target(s) [reference]	(q)PCR target for species differentiation [reference]
***Borrelia burgdorferi* sensu lato**	5S–23S IGS [30]	*pepX*, *recG* [43]
***Borrelia miyamotoi***	*flaB* [33]	*pepX*, *recG* [43]
***Anaplasma phagocytophilum***	*msp2* [34]	*groEL* [35]
***Rickettsia* spp.**	*gltA* [36]	*gltA* [44,45]
***Rickettsia helvetica***	*gltA* [37]	*gltA* [44,45]
***Neoehrlichia mikurensis***	*groEL* [38]	–
***Bartonella* spp.**	*ssrA* [39]	*gltA-1* [[46], [47], [48]]
***Spiroplasma* spp.**	*rpoB* [40]	–
***Babesia* spp. (excl. *B. microti*)**	*18S* [41]	*18S* [49]
***Babesia microti***	*ITS* [42]	*18S* [49]

### 2.4 Species differentiation of *Borrelia* spp., *Rickettsia* spp., *Bartonella* spp. and *Babesia* spp

Aliquots of *Borrelia* qPCR-positive samples were sent to the National Reference Center for *Borrelia* for further differentiation by multi-locus sequence typing (MLST). After DNA extraction using the Maxwell® 16 LEV Blood DNA Kit (Promega, Walldorf, Germany) according to manufacturer's recommendations, DNA concentrations were determined and samples diluted 1:2 or 1:5. All samples were re-tested by qPCR as described above [39]. Nested PCR to amplify fragments of the *pepX* and *recG* gene were carried out as previously described [52] with following modifications. We used a BioRad C1000 thermocycler and a PhireTaq mastermix (Invitrogen, ThermoFisher Scientific, Darmstadt, Germany). Primers and annealing temperatures were as described. Time for initial denaturation was 30 s and during each cycle 5 s; annealing time 5 s, elongation time 15 s. These conditions were used in first and second amplification rounds. Samples that showed bands of the expected size were sent for Sanger sequencing (GATC, Eurofins Genomics, Ebersberg, Germany).

*Rickettsia* positive samples were subjected to a semi-nested PCR amplifying a 382 bp part of the *gltA* gene with primers RpCs.780p, RpCS.1258n and RpCS.877p [53,54]. The 25 μl reaction contained 2.5 μl 10× buffer, 0.5 μl dNTPs (10 mM each), 0.5 μl of each primer (10 μM each), 0.25 μl DreamTaq® DNA polymerase (5 U/μl, Thermo Fisher Scientific Inc., Schwerte, Germany) and 5 μl template. In the second PCR, 0.5 μl of PCR product from the first round was used, and the amount of water was adjusted accordingly. The thermoprofile was performed as described by Ishikura et al. [54].

For species identification of *Bartonella*-positive samples, a 340 bp-fragment of the *gltA-1* gene was amplified using a semi-nested PCR protocol with primers 443f, 1210R and 781R [[55], [56], [57]]. The 25 μl reaction contained 2.5 μl 10× buffer, 1.5 μl MgCl_2_ (25 mM), 0.5 μl dNTPs (10 mM each), 0.5 μl of each primer (10 μM each), 0.25 μl HOT FIREPol® DNA polymerase (5 U/μl, Solis BioDyne, Tartu, Estonia) and 5 μl template. In the second PCR, 1 μl of PCR product from the first round was used, and the amount of water was adjusted accordingly. The thermoprofile included a 15 min polymerase activation step at 95 °C, followed by 40/25 cycles of 95 °C for 30 s, 53/56 °C for 30 s, 72 °C for 60 s in the first and the second round, respectively, and final elongation at 72 °C for 10 min.

*Babesia* qPCR-positive samples were subjected to a conventional PCR targeting the 18S rDNA by use of primers BJ1 and BN2 [58] with the reaction setup and thermoprofile described by Springer et al. [59].

After visualization on GelRed® (Biotium Inc., Fremont, CA, USA) stained 1.5% agarose gels, PCR products were custom Sanger sequenced (Microsynth Seqlab GmbH, Göttingen, Germany). Obtained sequences were compared to publicly available sequences using NCBI Blast.

### 2.5 Statistical analyses

To investigate the influence of temporal and animal-related factors on *Borrelia* seroprevalence as well as *Borrelia* spp. and *A. phagocytophilum* qPCR results, generalized linear models (GLMs) with binomial error structure and logit link were constructed in R v. 4.2.1 [60]. Sampling season (spring/summer/autumn/winter) and sampling year as well as animal sex and age category were included as covariates. For the model of *Borrelia* antibody seroprevalence, the corresponding *Borrelia* qPCR result was included as an additional covariate, while samples with a borderline ELISA result were excluded. In the models investigating qPCR results, a possible association between *Anaplasma* and *Borrelia* infection status was considered by including the respective other pathogen as a covariate. Furthermore, the weight of the cell pellet used for DNA extraction was included to control for a possible bias due to different sample amounts. For the remaining pathogens, no individual GLMs were calculated due to low prevalence. Full models were compared to null models including only the intercept in a likelihood ratio test.

## 3 Results

### 3.1 Sampled animals

A total of 541 hedgehogs were sampled, comprising 261 males (48.2%), 255 females (47.1%) and 25 animals of undetermined sex (4.6%). Approximately two-thirds of the animals were adult (63.6%, 344/541) and one-third were subadults (34.9%, 189/541), while the age category for eight animals (1.5%) remained undetermined. No juvenile hedgehogs were sampled. The ratio of adult to subadult animals varied throughout the year, with mostly adults sampled during spring (April–May; 70.8%, 119/168) and summer (June–August; 96.4%, 133/138) and mostly subadults during winter (December–February; 75.0%, 63/84), while the ratio was approximately even in autumn (51.7% subadults [78/151] vs. 48.3% adults [73/151]).

### 3.2 Serology results

Out of 541 hedgehogs, serological testing was performed on 539 for anti-*Borrelia* antibodies and 526 for anti-TBEV antibodies due to limited blood volume (Fig. 1). A significant majority, 84.8% (457/539), tested positive for *B. burgdorferi* s.l. antibodies, whereas 1.5% (8/539) yielded borderline results. Anti-TBEV antibodies were detected in one animal (0.2%) by ELISA and confirmed by SNT with a titre ≥1:20, while two further samples were considered borderline by ELISA but negative by SNT.

### 3.3 PCR results

A *Borrelia* qPCR analysis was performed on 539 hedgehogs, revealing a prevalence rate of 31.2% (168/539), as detailed in Table 2. The *Borrelia* 5S-23S IGS copy numbers were low, with a mean of 7.2 copies per reaction (median: 1.2). The 168 positive blood samples were sent to the National Reference Center for *Borrelia*, where additional DNA isolation and 5S-23S IGS qPCR were performed. A positive qPCR result was observed in 31 samples, with Ct values ranging between 35 and 40. *Borrelia* spp. differentiation by MLST was attempted, but no *Borrelia recG* nor *pepX* sequences could be generated. However, among the 525 samples tested additionally by *B. miyamotoi*-specific qPCR, one sample (0.2%) was positive for this species.

**Table 2:** Prevalence of vector-borne pathogens in blood samples from European hedgehogs in Northern Germany as determined by PCR.

Pathogen	Tested hedgehogs	Positive hedgehogs	Prevalence (%)	95% CI	Species differentiation
*Borrelia* spp.	539	168	31.4	27.3–35.3	Not successful
*Anaplasma phagocytophilum*	525	261	49.7	45.3–54.1	Ecotype I: 165/261 (63.2%); Ecotype II: 0/261 (0.0%); undetermined: 96/261 (36.8%)
*Rickettsia* spp.	525	42	8.0	5.8–10.7	*Rickettsia helvetica*: 21/42 (50.0%)^1^; *Rickettsia* sp.: 4/42 (9.5%)^1^; undetermined: 20/42 (47.6%)
*Neoehrlichia mikurensis*	525	0	0.0	0.0–0.01	NA
*Bartonella* spp.	525	68	13.0	10.2–16.1	*Bartonella* sp.: 50/68 (73.5%); undetermined: 18/68 (26.5%)
*Spiroplasma ixodetis*	525	43	8.2	6.0–10.9	NA
*Babesia* spp.	525	7	1.3	0.5–2.7	*Babesia microti*: 5/7 (71.4%), *Babesia venatorum*: 1/7 (14.3%), undetermined: 1/7 (14.3%)

1 Three hedgehogs were coinfected with *R. helvetica* and *Rickettsia* sp.

DNA of *A. phagocytophilum* was identified in 49.7% (261/525) of the hedgehog samples tested. Among these, 63.2% (165/261) were classified as ecotype I infections. The remaining positive samples did not match either ecotype I or II.

Prevalence of *Rickettsia* spp. amounted to 8.0% (42/525), with 50.0% (21/42) of infections assigned to *R. helvetica* by qPCR. Furthermore, a *Rickettsia gltA* sequence was obtained from four samples, while no *gltA* amplification was achieved for the remaining samples. All four sequences (GenBank acc. nos. PP104507-PP104510) showed 100% nucleotide identity to a *Rickettsia* sp. closely related to *Rickettsia felis* (99% query cover [QC], GenBank acc. nos. MG253006 and MG253007). Three of these samples were also positive for *R. helvetica* by qPCR.

Of the 68 *Bartonella*-positive samples (13.0%), 50 yielded *gltA-1* sequences (GenBank acc. nos. PP104457-PP104506) with 98.9–100% identity to a *Bartonella* sequence previously amplified from hedgehogs (100% QC, GenBank acc. no. MZ089843).

*Babesia* spp. DNA was detected in 1.3% (7/525) of samples, with five infections (GenBank acc. nos. PP097671, PP097672, PP097674-PP097676) determined as *B. microti* sensu stricto (s.s.) both by qPCR and by sequencing of the *18S* fragment (100% identity to *B. microti* HK strain [GenBank acc. no. AB085191], 100% QC), while one sample (GenBank acc. no. PP097673) yielded an 18S sequence that was 100% identical to published *Babesia venatorum* sequences (100% QC, e.g. GenBank acc. no. MT345317). Finally, *S. ixodetis* DNA was amplified from 8.2% of samples (43/525), while *N. mikurensis* was not detected.

### 3.4 Coinfections

Among the 525 hedgehogs tested for all pathogens, 70.1% (368/525) were positive for at least one pathogen, including 31.6% (166/525) that were coinfected with more than one pathogen. Single infections with *A. phagocytophilum* were detected in 22.1% (116/525), single infections with *Borrelia* spp. in 10.5% (55/525) and double infections with these two pathogens in 9.9% (52/525) of tested animals. Single infections with *Rickettsia* spp., *Bartonella* spp., *S. ixodetis* and *Babesia* spp. as well as various other coinfections with up to four different pathogens were each noted in <5% of hedgehogs (Table 3).

**Table 3:** Mono- and coinfections with vector-borne pathogens among 525 European hedgehogs.

Detected pathogen(s)^1^	Positive/Total	Prevalence (%)
*A. ph.* monoinfected	116/525	22.1
*Borr.* monoinfected	55/525	10.5
*Borr.* *+* *A. ph.*	52/525	9.9
*A. ph.* *+* *Spiropl.*	21/525	4.0
*Bart.* monoinfected	17/525	3.2
*A. ph.* *+* *Bart.*	17/525	3.2
*Borr.* *+* *A. ph.* *+* *Spiropl.*	13/525	2.5
*Rick.* monoinfected	12/525	2.3
*Borr.* *+* *Bart.*	11/525	2.1
*Borr.* *+* *A. ph.* *+* *Bart.*	11/525	2.1
*Borr.* *+* *A. ph.* *+* *Rick.*	9/525	1.7
*A. ph.* *+* *Rick.*	7/525	1.3
*Borr.* *+* *Rick.*	6/525	1.1
*A. ph.* *+* *Bab.*	3/525	0.6
*A. ph.* *+* *Rick.* *+* *Bart.*	2/525	0.4
*A. ph.* *+* *Spiropl.* *+* *Bart.*	2/525	0.4
*Borr.* *+* *A. ph.* *+* *Rick.* *+* *Bart.*	2/525	0.4
*Borr.* *+* *A. ph.* *+* *Spiropl.* *+* *Bart.*	2/525	0.4
Other^2^	10/525	1.9

1 Abbreviations: *A. ph.*, *A. phagocytophilum*; *Borr.*, *Borrelia* spp.; *Bab.*, *Babesia* spp.; *Bart*., *Bartonella* spp.; *Rick.*, *Rickettsia* spp.; *Spiropl.*, *Spiroplasma* spp.

2 One animal each infected with *Bab.* only, *Spiropl.* only, *Bab.* *+* *Bart., Spiropl.* *+* *Bart.*, *Spiropl.* *+* *Rick.*, *Borr.* *+* *A. ph.* *+* *Bab.*, *A. ph.* *+* *Bab.* *+* *Bart.*, *A. ph.* *+* *Spiropl.* *+* *Rick.*, *Borr.* *+* *Rick.* *+* *Bart.*, *Borr.* *+* *A. ph.* *+* *Spiropl.* *+* *Rick.*

### 3.5 Temporal and host-related patterns

**Fig. 2:**
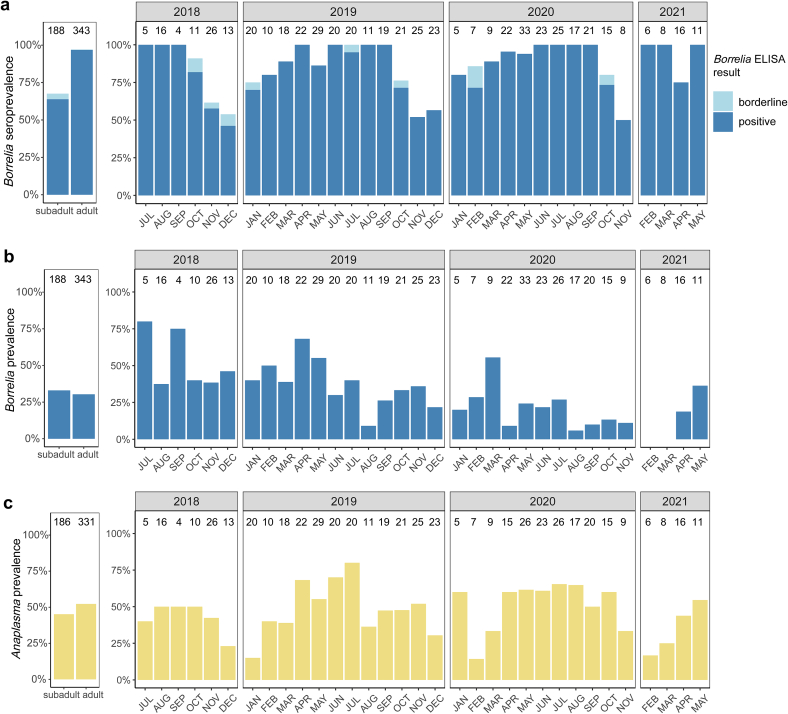
Patterns of (a) *B. burgdorferi* s.l. ELISA results, (b) *Borrelia* spp. qPCR results and (c) *A. phagocytophilum* qPCR results in blood samples of European hedgehogs according to animal age class and over the course of the study. Sample sizes are indicated above the bars. Note that animals with undetermined age class were excluded from the panels on the left. No sampling was done in December 2020 and January 2021.

The seroprevalence of *Borrelia* antibodies showed a seasonal pattern with a higher proportion of seropositive animals in the summer months. However, the GLM indicated that this was driven by the age distribution of the animals, with a significantly higher probability of seropositivity in adults, which were predominantly sampled during summer (Fig. 2, Table 4). In contrast, animal sex, season, sampling year and *Borrelia* qPCR result were not significantly associated with *Borrelia* antibody status. In fact, among *Borrelia* seropositive animals, 32.6% (149/457) were positive by qPCR, while 24.3% (17/74) of seronegative animals were also qPCR-positive (Table 5).

**Table 4:** Results of the generalized linear model investigating the influence of temporal and animal-related factors on *Borrelia* antibody status in 496⁎ European hedgehogs. The full model was significantly different from a null model containing only the intercept (χ^2^ = 109.44, Df = 9, *P* < 0.001).

	Estimate	Std. Error	z-value	*P*-value
Intercept	0.48	0.49	0.99	0.321
Season (ref.: winter)				
Spring	0.42	0.45	0.92	0.355
Summer	16.73	937.54	0.02	0.986
Autumn	−0.33	0.37	−0.88	0.378
Sampling year (ref.: 2018)				
2019	−0.12	0.43	−0.28	0.777
2020	0.15	0.53	0.29	0.775
2021	0.27	0.81	0.33	0.743
Age (adult vs. subadult)	2.28	0.40	5.64	**< 0.001**
Sex (male vs. female)	0.18	0.31	0.59	0.556
*Borrelia* qPCR result (positive vs. negative)	0.49	0.34	1.43	0.152

⁎ Samples with a borderline ELISA result were excluded.

**Table 5:** Prevalence of *Borrelia* spp. DNA in blood samples of European hedgehogs from northern Germany according to their anti-*Borrelia* antibody status.

	ELISA result			
	Seropositive (*N* = 457)	Borderline (*N* = 8)	Seronegative (*N* = 74)	Not tested by ELISA (*N* = 2)
***Borrelia* qPCR-positive**	149/457 (32.6%)	1/8 (12.5%)	18/74 (24.3%)	0 (0.0%)
***Borrelia* qPCR-negative**	306/457 (67.0%)	7/8 (87.5%)	56/74 (75.7%)	2 (100%)
**Not tested by qPCR**	2/457 (0.4%)	0/8 (0.0%)	0 (0.0%)	NA

**Supplementary Fig. S1:**
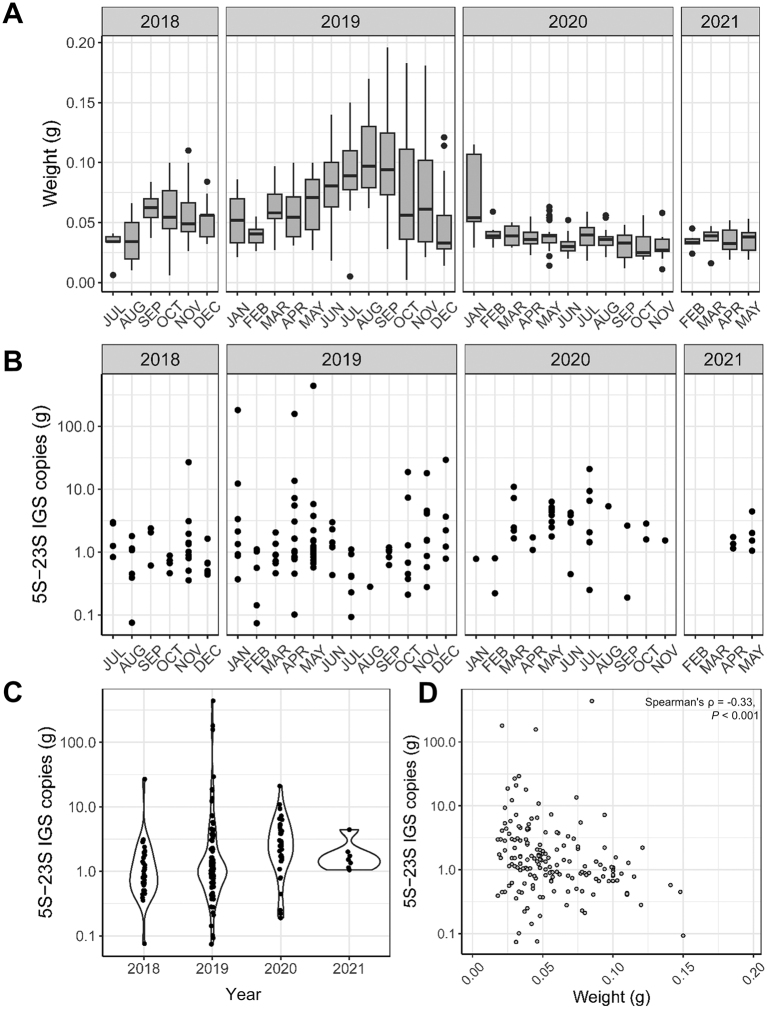
Amount of blood clot used for isolation of DNA from hedgehog blood samples (A) and Borrelia 5S-23S intergenic spacer (IGS) copies (B) in positive samples over the course of the study. Violin plots (C) illustrate the shape of the 5S-23S IGS copy number distribution in the different study years, with individual values shown as dots. The correlation between blood clot weight and 5S-23S IGS copy numbers is shown in panel D.

Regarding *Borrelia* spp. infection status as determined by qPCR, no significant age, sex or seasonal difference was determined, whereas a decline in prevalence was noted over the course of the study (Fig. 2, Table 6). However, a lower amount of blood was used on average for DNA extraction in 2020 and 2021 than in 2018 and 2019 (Supplementary Fig. S1). Although this covariate did not show a significant influence in the GLM for *Borrelia* prevalence (Table 6), a higher amount of blood was significantly associated with lower *Borrelia* copy numbers among positive samples (Figure Supplementary Fig. S1).

In contrast, no decline in prevalence was apparent for *A. phagocytophilum*. For this pathogen, a seasonal pattern was observed with a significantly higher prevalence in spring, summer and autumn as compared to winter, whereas the model indicated no significant age or sex effect (Fig. 2, Table 6). No significant association between *Borrelia* and *Anaplasma* infection status was observed.

**Table 6:** Results of the generalized linear models investigating the influence of temporal and animal-related factors on *Borrelia* and *Anaplasma* infection status as determined by qPCR in 492 European hedgehogs. Significant *P*-values are printed in bold. The full models were significantly different from null models containing only the intercept (model A: χ^2^ = 37.8, Df = 10, *P* < 0.001; model B: χ^2^ = 32.9, Df = 10, *P* < 0.001).

	Model A: *Borrelia* spp. prevalence				Model B: *Anaplasma* spp. prevalence			
	Estimate	Std. Error	z-value	*P*-value	Estimate	Std. Error	z-value	*P*-value
Intercept	−0.32	0.39	−0.82	0.411	−1.45	0.41	−3.57	**< 0.001**
Season (ref.: winter)								
Spring	0.53	0.35	1.55	0.122	1.13	0.33	3.37	**0.001**
Summer	−0.35	0.40	−0.89	0.373	1.55	0.37	4.24	**< 0.001**
Autumn	−0.23	0.34	−0.70	0.485	0.92	0.32	2.88	**0.004**
Sampling year (ref.: 2018)								
2019	−0.40	0.32	−1.25	0.212	0.28	0.32	0.88	0.378
2020	−1.44	0.35	−4.07	**< 0.001**	0.58	0.33	1.75	0.079
2021	−1.78	0.55	−3.25	**0.001**	−0.07	0.50	−0.14	0.888
Age (adult vs. subadult)	0.11	0.26	0.43	0.667	−0.30	0.24	−1.27	0.205
Sex (male vs. female)	0.05	0.21	0.27	0.790	−0.01	0.19	−0.07	0.947
*Anaplasma* qPCR result (positive vs. negative)	0.39	0.21	1.84	0.066	–	–	–	–
*Borrelia* qPCR result (positive vs. negative)	–	–	–	–	0.38	0.21	1.82	0.069
Amount of blood clot used for DNA isolation	−1.03	3.65	−0.28	0.778	3.56	3.45	1.03	0.303

**Fig. 3:**
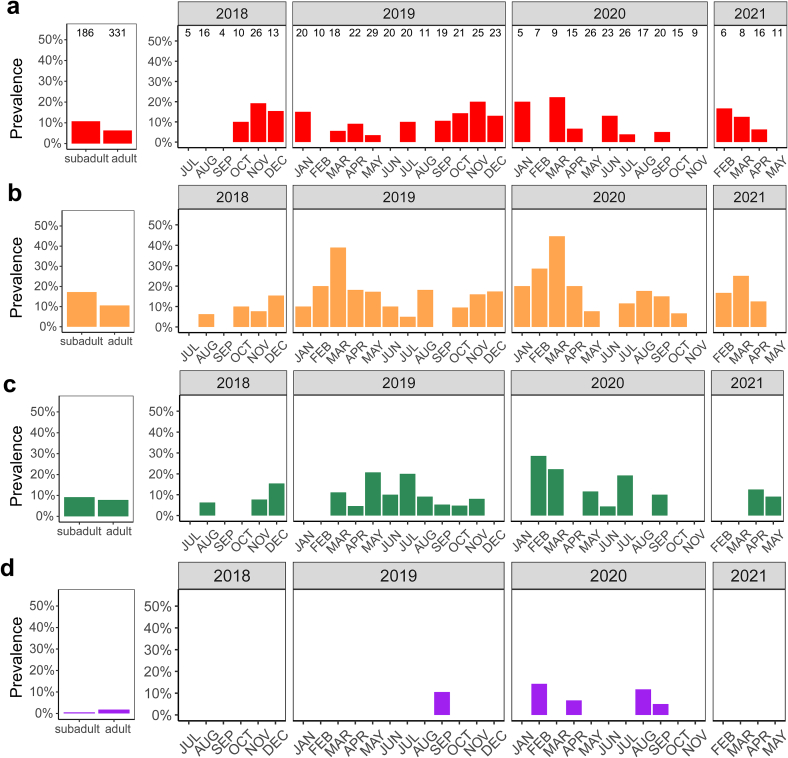
Prevalence of (a) *Rickettsia* spp., (b) *Bartonella* spp., (c) *Spiroplasma ixodetis* and (d) *Babesia* spp. in blood samples of European hedgehogs according to animal age class and over the course of the study. Sample sizes are indicated above the bars of panel A and refer to all panels. Note that animals with undetermined age class were excluded from the panels on the left. No sampling was done in December 2020 and January 2021.

For the remaining pathogens, detection rates in the two age groups as well as over the course of the study are visualized in Fig. 3, but no statistical models were calculated due to the low prevalence values.

## 4 Discussion

Hedgehogs often show high levels of ectoparasite infestation [24,25]. Therefore, the high prevalence and species diversity of vector-borne pathogens detected in the present study was not unexpected. The fact that almost all adult animals, i.e. animals >1 year, had antibodies against *B. burgdorferi* s.l. confirms a high level of exposure. In contrast, neutralizing antibodies against TBEV were detected in one animal only. Most parts of Northern Germany, including the region of Hanover, are not regarded as TBEV risk areas [61] and the distribution of TBEV is restricted to small foci even within endemic regions [62]. This and the rather small home range size of hedgehogs compared to larger wildlife species may explain the low TBEV seroprevalence.

Moreover, hedgehog blood samples were screened for several vector-borne pathogens by qPCR. No tissue samples were taken for ethical reasons, as it was the goal to rehabilitate the hedgehogs which were often weak and/or injured. Blood is not regarded as an ideal sample type for *B. burgdorferi* s.l. detection because spirochetemia is usually transient and low-level [63,64]. For example, *B. burgdorferi* was cultured approximately ten times more often from the spleen than from blood of white-footed mice (*Peromyscus leucopus*) [65]. Therefore, the rather high rate of 31.2% *Borrelia* spp.-positive blood samples in the present study was surprising, but may be due to a high sensitivity of the employed probe-based qPCR. In a previous study based on tissue samples of European hedgehogs from the Czech Republic, a 90.0% detection rate of *B. burgdorferi* s.l. DNA was reported [66], in line with the high seroprevalence rate determined in the present study. In contrast, another study, also based on hedgehog tissue samples, reported only a 13.5% detection rate [67]. This discrepancy may be due to differences in the sensitivity of employed PCR techniques, the tissue sample types, regional differences or temporal fluctuations.

Interestingly, the *Borrelia* spp. detection rate declined significantly over the course of the present study. However, this result should be treated with caution as the amount of blood used for DNA isolation was on average lower in 2020 and 2021 (0.04 g) than in 2018 and 2019 (0.07 g) to avoid clogging of DNA isolation columns. Although no statistically significant effect of the amount of blood on the *Borrelia* detection rate could be discerned, the *Borrelia* 5S-23S IGS copy numbers were negatively correlated with the weight of the blood clot, which could mean that a higher amount of blood allowed detection of lower copy numbers. Thus, infections with a low level of spirochetemia may have been missed in 2020 and 2021 due to the lower amount of blood used.

A significant seasonal pattern was neither detected for *Borrelia* ELISA nor for qPCR results, as the apparently higher antibody detection rate in summer was driven by the high proportion of adult animals sampled during these months. The age difference in the seropositivity rate can be attributed to cumulative exposure combined with a long-lived antibody response. The fact that animals were qPCR positive despite being antibody-positive, without any seasonal pattern, implies that they may be more persistent carriers than e.g. rodents, which show low infection rates during winter [68,69]. As hedgehogs also move larger distances than rodents, this indicates a potentially important role for pathogen maintenance and spread, especially in urban areas, where tick density can be low.

To further elucidate the zoonotic potential of the *Borrelia* spp. harboured by hedgehogs, species differentiation by MLST was attempted but was not successful, probably due to the very low amount of borrelial DNA in the samples, whereas only one sample was *B. miyamotoi*-positive by qPCR. In previous studies, *B. afzelii*, *B. garinii*, *B. bavariensis*, *B. spielmanii*, as well as *B. miyamotoi,* among others, were all detected in European hedgehogs, with most infections attributed to *B. afzelii* [35,66,67]. Moreover, *B. afzelii*, *B. bavariensis* and *B. spielmanii* were detected significantly more often in engorged ticks from European hedgehogs than in questing ticks in a Dutch study, indicating that hedgehogs may serve as a reservoir for these species [25].

Furthermore, hedgehogs are regarded as potential reservoirs for *A. phagocytophilum* [34,70]. In the present study, *A. phagocytophilum* was the most frequently detected pathogen, with a prevalence of almost 50%. A higher detection rate of >90% was reported in skin biopsies of European hedgehogs from the Czech Republic, while the prevalence in blood samples amounted to 75.0% [70]. Furthermore, a study based on repeated blood sampling of a captive, but tick-exposed hedgehog population in Germany determined monthly prevalence values between 41.7 and 84.6% [34]. Although it should be kept in mind that the hedgehogs in the mentioned study did not live under completely natural conditions, one interesting fact was that individual animals showed repeated bouts of bacteraemia, with highest detection rates in spring and autumn. A seasonal pattern in *A. phagocytophilum* prevalence was also apparent in the present study, with higher detection rates from spring through autumn compared to winter, probably related to the main activity period of *I. ricinus* in central Europe [71].

European hedgehogs may carry several ecotypes of *A. phagocyophilum*, including zoonotic variants [70,72,73].Approximately two-thirds of the *A. phagocytophilum* infections in the present study were attributed to ecotype 1. This ecotype, also referred to as cluster 1 based on MLST and *ankA* phylogenies [72], is associated with *I. ricinus* ticks and constitutes the most frequent ecotype detected in European as well as Northern white-breasted hedgehogs [35,70,74]. Moreover, almost all isolates from hedgehogs form a monophyletic group with zoonotic strains within ecotype 1/cluster 1 [reviewed by 72], emphasizing the reservoir role of this wildlife species for human-pathogenic *A. phagocytophilum*. Like ecotype 1, ecotype 2 is associated with *I. ricinus* ticks, but mainly with ruminants as reservoirs [74]. This ecotype was not detected in the present study. The remaining undifferentiated *A. phagocytophilum* infections may have included ecotype 4, which is mainly associated with birds [74], but has recently also been detected in European hedgehogs [70].

With a prevalence of 13%, *Bartonella* spp. were the third most frequently detected pathogens in the present study. An even higher prevalence of 24% was noted in European hedgehogs in the Czech Republic [36]. This difference may again be due to the use of blood instead of tissue samples in the present study, as Majerová et al. [36] determined higher infection rates in spleen and ear samples than in blood. *Bartonella* spp. differentiation was successful in >70% of cases, yielding a *gltA* sequence previously amplified from European and Northern white-breasted hedgehogs [36]. No other *Bartonella* spp. were detected, in contrast to previous findings of *Bartonella washoensis*, *Bartonella melophagi* and another undescribed species, *Bartonella* sp. SCIER [36]. It remains unclear whether the identified *Bartonella* sp., which seems closely related to the cat-associated zoonotic pathogen *Bartonella clarridgeiae* [36], is tick- or flea-transmitted and whether it has any clinical significance for humans or domestic animals.

In contrast to the rather high prevalence values of *Borrelia* spp., *A. phagocytophilum* and *Bartonella* spp., the prevalence of *Rickettsia* spp. amounted to only 8.0%, which seems especially low in light of high detection rates in questing ticks in Northern Germany, e.g. 50.8% in the city of Hanover in 2015 [75] and 36.0% in 2020 [76]. In other north-western German areas, similar values between 22.4 and 36.5% *Rickettsia*-positive ticks were recorded, with the vast majority of infections attributed to *R. helvetica* [77]. In the present study, *R. helvetica* was identified in 50.0% of *Rickettsia*-positive hedgehogs by qPCR. While no comparable studies on *Rickettsia* infections of European hedgehogs have been published to the authors' knowledge, reports exist of *R. helvetica* in different rodent species (e.g. [78,79]) and Northern white-breasted hedgehogs [35]. However, transmission of this rickettsial species to vertebrates does not seem to be very efficient, or results only in a very short-lived rickettsemia. For example, *R. helvetica* was detected in approximately 10% of immature ticks collected from 158 rodents in Poland, but not in the rodent blood samples [80]. Similarly, *R. helvetica* prevalence in blood samples of birds amounted to only 4.7%, but was tenfold higher in ticks collected from these birds [81]. Like other spotted-fever group rickettsiae, *R. helvetica* can be regarded as a tick symbiont with efficient vertical transmission in the tick population [82], which may explain its low propensity to cause systemic infection in vertebrates.

Because hedgehogs are frequently infested with fleas, infection with flea-associated *Rickettsia* spp. seems likely. *Rickettsia felis* and a closely related *Rickettsia* sp. have been detected in the hedgehog flea *Archaeopsylla erinacei* at high prevalence [83,84]. Unfortunately, further differentiation of *Rickettsia* positive samples from the current study by sequencing of the *gltA* gene was only successful in four cases, probably due to the low amount of *Rickettsia* DNA indicated by high Ct values. The amplified *gltA* sequences were 100% identical to a *Rickettsia* sp. closely related to *R. felis*, which was previously detected in yellow-necked mice (*Apodemus flavicollis*) in Slovakia [85].

*Neoehrlichia mikurensis* is another member of the order Rickettsiales which has previously been detected in Northern white-breasted hedgehogs at a low prevalence of 2.3% [33]. It was not detected in the present study, in contrast to high detection rates in blood samples of various rodents in different European countries [86,87]. Therefore, European hedgehogs may not be suitable reservoir hosts for this pathogen. This is also supported by the fact that *N. mikurensis* was not detected at higher prevalence in engorged ticks from hedgehogs than in questing ticks, contrary to different *B. burgdorferi* s.l. species and *A. phagocytophilum* [25].

Moreover, DNA of *S. ixodetis*, an endosymbiont of ticks and other arthropods [88], was detected in 8.2% of hedgehogs and thus at a similar frequency as *Rickettsia* species. Like *Rickettsia* spp., *S. ixodetis* is transmitted vertically in *Ixodes* populations [89], and transmission to hedgehogs may be incidental and without epidemiological relevance. Nevertheless, the detection in hedgehogs indicates potential exposure of humans in the study region, which is of interest as *S. ixodetis* is suspected to be human pathogenic under certain conditions [16].

Concerning tick-transmitted protozoa, a 1.3% prevalence of *Babesia* spp. was noted. Five of seven infections were attributed to rodent-associated *B. microti* and one to *B. venatorum*, while the *Babesia* sp. in the remaining qPCR-positive sample could not be identified. The generated 18S sequences corresponded to *B. microti* s.s. (clade 1), which is responsible for most human babesiosis cases [90] and associated with different rodents and shrews as reservoir hosts [[91], [92], [93]]. To the authors' knowledge, this is the first report of *B. microti* in European hedgehogs. The detection of *B. venatorum* in a European hedgehog was even more surprising, as this species is associated with roe deer, although it has also been found in sheep [94]. As the molecular assay does not allow to conclude whether the amplified DNA in the present study belonged to viable parasites, it remains questionable whether the hedgehog was truly infected or only carried DNA of *B. venatorum* in its bloodstream due to the bite of an infected tick.

## 5 Conclusions

The present study showed that hedgehogs from wildlife rescue centres provide a valuable source of information on the occurrence of One Health pathogens in (peri-)urban areas. These hedgehogs can be sampled during initial clinical examination, without the necessity of trapping and even killing them. A high species diversity of vector-borne pathogens and a particularly high prevalence of *Borrelia* spp. and *A. phagocytophilum* was detected among the European hedgehogs in the present study, which puts this species in the spotlight of potential reservoir hosts for the most important zoonotic VBDs in urban areas. Moreover, the fact that more than a third of hedgehogs carried coinfections indicates that hedgehogs may be an important source of coinfections in ticks, which poses an increased health risk to humans due to increased disease severity and diagnostic challenges [95]. As hedgehogs are expected to thrive in urban environments as a consequence of increased urban greening, the risk for VBD transmission may increase. Municipalities should be aware of this risk and act to raise awareness among citizens and veterinary and medical doctors to improve preventive measures and enhance VBD diagnosis. For example, as domestic dogs and particularly free-ranging cats are prone to roam in hedgehog habitats, protecting them against ectoparasites is equally important for the sake of their health and to avoid translocation of ticks and fleas into human households. When hedgehogs are taken into human care, they should be immediately treated against ectoparasites to avoid transfer of potentially infected vectors to human caretakers. Further studies are necessary to quantify hedgehog density in different environments and to compare their VBD infection rates between urban and rural areas, calling for close collaboration between veterinarians, ecologists, public health professionals, and conservationists.

The following is the supplementary data related to this article.

## Funding statement

This study was financially supported by a grant to KS of the Grimminger Stiftung für Zoonosenforschung and by a grant of the European Union through the 10.13039/501100008530European Regional Development Fund and the Interreg North Sea Region Programme 2014–2020 as part of the NorthTick project (reference number J-No.: 38–2–7–19).

## CRediT authorship contribution statement

**Andrea Springer:** Investigation, Visualization, Writing – original draft. **Karolin Schütte:** Investigation, Writing – review & editing. **Florian Brandes:** Funding acquisition, Supervision, Writing – review & editing. **Maximilian Reuschel:** Investigation, Writing – review & editing. **Michael Fehr:** Supervision, Writing – review & editing. **Gerhard Dobler:** Investigation, Writing – review & editing. **Gabriele Margos:** Investigation, Writing – review & editing. **Volker Fingerle:** Resources, Writing – review & editing. **Hein Sprong:** Funding acquisition, Investigation, Writing – review & editing. **Christina Strube:** Conceptualization, Funding acquisition, Supervision, Writing – review & editing.

## Declaration of competing interest

The authors declare that they have no conflicts of interest.

## Data Availability

Data supporting reported results is contained within the article. Sequences generated during this study have been submitted to NCBI Genbank under accession nos. PP097671-PP097676 and PP104457-PP104510.

## References

[1] Combs M.A., Kache P.A., VanAcker M.C., Gregory N., Plimpton L.D., Tufts D.M., Fernandez M.P., Diuk-Wasser M.A. (2022). Socio-ecological drivers of multiple zoonotic hazards in highly urbanized cities. Glob. Chang. Biol..

[2] Murray M.H., Sánchez C.A., Becker D.J., Byers K.A., Worsley-Tonks K.E., Craft M.E. (2019). City sicker? A meta-analysis of wildlife health and urbanization. Front. Ecol. Environ..

[3] Heaviside C., Macintyre H., Vardoulakis S. (2017). The urban Heat Island: implications for health in a changing environment. Current Environmental Health Rep..

[4] Coutts C., Hahn M. (2015). Green infrastructure, ecosystem services, and human health. Int. J. Environ. Res. Public Health.

[5] Heylen D., Lasters R., Adriaensen F., Fonville M., Sprong H., Matthysen E. (2019). Ticks and tick-borne diseases in the city: role of landscape connectivity and green space characteristics in a metropolitan area. Sci. Total Environ..

[6] Mackenstedt U., Jenkins D., Romig T. (2015). The role of wildlife in the transmission of parasitic zoonoses in peri-urban and urban areas. Int. J. Parasitol. Parasites Wildl..

[7] Semenza J.C., Suk J.E. (2017). Vector-borne diseases and climate change: a European perspective. FEMS Microbiol. Lett..

[8] Bajer A., Beck A., Beck R., Behnke J.M., Dwużnik-Szarek D., Eichenberger R.M., Farkas R., Fuehrer H.-P., Heddergott M., Jokelainen P., Leschnik M., Oborina V., Paulauskas A., Radzijevskaja J., Ranka R., Schnyder M., Springer A., Strube C., Tolkacz K., Walochnik J. (2022). Babesiosis in southeastern, central and northeastern Europe: an emerging and re-emerging tick-borne disease of humans and animals. Microorganisms.

[9] Mysterud A., Jore S., Østerås O., Viljugrein H. (2017). Emergence of tick-borne diseases at northern latitudes in Europe: a comparative approach. Sci. Rep..

[10] Beugnet F., Marié J.-L. (2009). Emerging arthropod-borne diseases of companion animals in Europe. Vet. Parasitol..

[11] Burn L., Tran T.M.P., Pilz A., Vyse A., Fletcher M.A., Angulo F.J., Gessner B.D., Moïsi J.C., Jodar L., Stark J.H. (2023). Incidence of Lyme Borreliosis in Europe from national surveillance systems (2005–2020). Vector Borne Zoonot. Dis..

[12] Brzozowska M., Wierzba A., Śliwczyński A., Myśliwiec M., Kozłowski K., Wierzba W. (2021). The problem of Lyme borreliosis infections in urban and rural residents in Poland, based on National Health Fund data. Ann. Agric. Environ. Med..

[13] Linard C., Lamarque P., Heyman P., Ducoffre G., Luyasu V., Tersago K., Vanwambeke S.O., Lambin E.F. (2007). Determinants of the geographic distribution of Puumala virus and Lyme borreliosis infections in Belgium. Int. J. Health Geogr..

[14] Mac S., da Silva S.R., Sander B. (2019). The economic burden of Lyme disease and the cost-effectiveness of Lyme disease interventions: a scoping review. PLoS One.

[15] Sprong H., Azagi T., Hoornstra D., Nijhof A.M., Knorr S., Baarsma M.E., Hovius J.W. (2018). Control of Lyme borreliosis and other *Ixodes ricinus*-borne diseases. Parasit. Vectors.

[16] Eimer J., Fernström L., Rohlén L., Grankvist A., Loo K., Nyman E., Henningsson A.J., Haglund M., Hultqvist V., Sjöwall J., Wennerås C., Schön T. (2022). *Spiroplasma ixodetis* infections in immunocompetent and immunosuppressed patients after tick exposure, Sweden. Emerg. Infect. Dis..

[17] Wolcott K.A., Margos G., Fingerle V., Becker N.S. (2021). Host association of *Borrelia burgdorferi* sensu lato: a review. Ticks Tick Borne Dis..

[18] Rizzoli A., Silaghi C., Obiegala A., Rudolf I., Hubálek Z., Földvári G., Plantard O., Vayssier-Taussat M., Bonnet S., Špitalská E., Kazimírová M. (2014). *Ixodes ricinus* and its transmitted pathogens in urban and peri-urban areas in Europe: new hazards and relevance for public health. Front. Public Health.

[19] Hubert P., Julliard R., Biagianti S., Poulle M.-L. (2011). Ecological factors driving the higher hedgehog (*Erinaceus europeaus*) density in an urban area compared to the adjacent rural area. Landsc. Urban Plan..

[20] van de Poel J.L., Dekker J., van Langevelde F. (2015). Dutch hedgehogs *Erinaceus europaeus* are nowadays mainly found in urban areas, possibly due to the negative effects of badgers *Meles meles*. Wildl. Biol..

[21] Gazzard A., Yarnell R.W., Baker P.J. (2022). Fine-scale habitat selection of a small mammalian urban adapter: the West European hedgehog (*Erinaceus europaeus*). Mamm. Biol..

[22] Bearman-Brown L.E., Baker P.J. (2022). An estimate of the scale and composition of the hedgehog (*Erinaceus europaeus*) rehabilitation community in Britain and the Channel Islands. Animals.

[23] Lukešová G., Voslarova E., Vecerek V., Vucinic M. (2021). Trends in intake and outcomes for European hedgehog (*Erinaceus europaeus*) in the Czech rescue centers. PLoS One.

[24] Schütte K., Springer A., Brandes F., Reuschel M., Fehr M., Strube C. (2024). Ectoparasites of European hedgehogs (*Erinaceus europaeus*) in Germany and their health impact. Parasit. Vectors.

[25] Jahfari S., Ruyts S.C., Frazer-Mendelewska E., Jaarsma R., Verheyen K., Sprong H. (2017). Melting pot of tick-borne zoonoses: the European hedgehog contributes to the maintenance of various tick-borne diseases in natural cycles urban and suburban areas. Parasit. Vectors.

[26] Mihalca A., Sándor A. (2013). The role of rodents in the ecology of *Ixodes ricinus* and associated pathogens in Central and Eastern Europe. Front. Cell. Infect. Microbiol..

[27] Gern L., Toutoungi L.N., Hu C.M., Aeschlimann A. (1991). *Ixodes* (*Pholeoixodes*) *hexagonus*, an efficient vector of *Borrelia burgdorferi* in the laboratory. Med. Vet. Entomol..

[28] Toutoungi L.N., Gern L. (1993). Ability of transovarially and subsequent transstadially infected *Ixodes hexagonus* ticks to maintain and transmit *Borrelia burgdorferi* in the laboratory. Exp. Appl. Acarol..

[29] Springer A., Raulf M.-K., Fingerle V., Strube C. (2020). *Borrelia* prevalence and species distribution in ticks removed from humans in Germany, 2013–2017. Ticks Tick Borne Dis..

[30] Lernout T., De Regge N., Tersago K., Fonville M., Suin V., Sprong H. (2019). Prevalence of pathogens in ticks collected from humans through citizen science in Belgium. Parasit. Vectors.

[31] Probst J., Springer A., Strube C. (2023). Year-round tick exposure of dogs and cats in Germany and Austria: results from a tick collection study. Parasit. Vectors.

[32] Gern L., Rouvinez E., L.N. Toutoungi, Godfroid E. (1997). Transmission cycles of *Borrelia burgdorferi* sensu lato involving *Ixodes ricinus* and/or *I. hexagonus* ticks and the European hedgehog, *Erinaceus europaeus*, in suburban and urban areas in Switzerland. Folia Parasitol..

[33] Földvári G., Jahfari S., Rigó K., Jablonszky M., Szekeres S., Majoros G., Tóth M., Molnár V., Coipan E.C., Sprong H. (2014). *Candidatus* Neoehrlichia mikurensis and *Anaplasma phagocytophilum* in urban hedgehogs. Emerg. Infect. Dis..

[34] Silaghi C., Skuballa J., Thiel C., Pfister K., Petney T., Pfäffle M., Taraschewski H., Passos L.M.F. (2012). The European hedgehog (*Erinaceus europaeus*) – a suitable reservoir for variants of *Anaplasma phagocytophilum*?. Ticks Tick Borne Dis..

[35] Szekeres S., Docters van Leeuwen A., Tóth E., Majoros G., Sprong H., Földvári G. (2019). Road-killed mammals provide insight into tick-borne bacterial pathogen communities within urban habitats. Transbound. Emerg. Dis..

[36] Majerová K., Gutiérrez R., Fonville M., Hönig V., Papežík P., Hofmannová L., Lesiczka P.M., Nachum-Biala Y., Růžek D., Sprong H., Harrus S., Modrý D., Votýpka J. (2021). Hedgehogs and squirrels as hosts of zoonotic *Bartonella* species. Pathogens.

[37] Okaro U., Addisu A., Casanas B., Anderson B. (2017). *Bartonella* species, an emerging cause of blood-culture-negative endocarditis. Clin. Microbiol. Rev..

[38] Girl P., Haut M., Riederer S., Pfeffer M., Dobler G. (2021). Comparison of three serological methods for the epidemiological investigation of TBE in dogs. Microorganisms.

[39] Strube C., Montenegro V.M., Epe C., Eckelt E., Schnieder T. (2010). Establishment of a minor groove binder-probe based quantitative real time PCR to detect *Borrelia burgdorferi* sensu lato and differentiation of *Borrelia spielmanii* by ospA-specific conventional PCR. Parasit. Vectors.

[40] Tappe J., Jordan D., Janecek E., Fingerle V., Strube C. (2014). Revisited: *Borrelia burgdorferi* sensu lato infections in hard ticks (*Ixodes ricinus*) in the city of Hanover (Germany). Parasit. Vectors.

[41] May K., Jordan D., Fingerle V., Strube C. (2015). *Borrelia burgdorferi* sensu lato and co-infections with *Anaplasma phagocytophilum* and *Rickettsia* spp. in *Ixodes ricinus* in Hamburg, Germany. Med. Vet. Entomol..

[42] Hovius J.W.R., de Wever B., Sohne M., Brouwer M.C., Coumou J., Wagemakers A., Oei A., Knol H., Narasimhan S., Hodiamont C.J., Jahfari S., Pals S.T., Horlings H.M., Fikrig E., Sprong H., van Oers M.H.J. (2013). A case of meningoencephalitis by the relapsing fever spirochaete *Borrelia miyamotoi* in Europe. Lancet.

[43] Courtney J.W., Kostelnik L.M., Zeidner N.S., Massung R.F. (2004). Multiplex real-time PCR for detection of *Anaplasma phagocytophilum* and *Borrelia burgdorferi*. J. Clin. Microbiol..

[44] Gandy S., Hansford K., McGinley L., Cull B., Smith R., Semper A., Brooks T., Fonville M., Sprong H., Phipps P., Johnson N., Medlock J.M. (2022). Prevalence of *Anaplasma phagocytophilum* in questing *Ixodes ricinus* nymphs across twenty recreational areas in England and Wales. Ticks Tick Borne Dis..

[45] Stenos J., Graves S.R., Unsworth N.B. (2005). A highly sensitive and specific real-time PCR assay for the detection of spotted fever and typhus group Rickettsiae. Am. J. Trop. Med. Hyg..

[46] Heylen D., Fonville M., van Leeuwen A.D., Sprong H. (2016). Co-infections and transmission dynamics in a tick-borne bacterium community exposed to songbirds. Environ. Microbiol..

[47] Jahfari S., Fonville M., Hengeveld P., Reusken C., Scholte E.-J., Takken W., Heyman P., Medlock J.M., Heylen D., Kleve J. (2012). Prevalence of *Neoehrlichia mikurensis* in ticks and rodents from North-West Europe. Parasit. Vectors.

[48] Diaz M.H., Bai Y., Malania L., Winchell J.M., Kosoy M.Y. (2012). Development of a novel genus-specific real-time PCR assay for detection and differentiation of *Bartonella* species and genotypes. J. Clin. Microbiol..

[49] Subramanian G., Sekeyova Z., Raoult D., Mediannikov O. (2012). Multiple tick-associated bacteria in *Ixodes ricinus* from Slovakia. Ticks Tick Borne Dis..

[50] Øines Ø., Radzijevskaja J., Paulauskas A., Rosef O. (2012). Prevalence and diversity of *Babesia* spp. in questing *Ixodes ricinus* ticks from Norway. Parasit. Vectors.

[51] Kazimírová M., Hamšíková Z., Špitalská E., Minichová L., Mahríková L., Caban R., Sprong H., Fonville M., Schnittger L., Kocianová E. (2018). Diverse tick-borne microorganisms identified in free-living ungulates in Slovakia. Parasit. Vectors.

[52] Margos G., Gatewood A.G., Aanensen D.M., Hanincová K., Terekhova D., Vollmer S.A., Cornet M., Piesman J., Donaghy M., Bormane A., Hurn M.A., Feil E.J., Fish D., Casjens S., Wormser G.P., Schwartz I., Kurtenbach K. (2008). MLST of housekeeping genes captures geographic population structure and suggests a European origin of *Borrelia burgdorferi*. Proc. Natl. Acad. Sci. USA.

[53] Regnery R.L., Spruill C.L., Plikaytis B.D. (1991). Genotypic identification of rickettsiae and estimation of intraspecies sequence divergence for portions of two rickettsial genes. J. Bacteriol..

[54] Ishikura M., Ando S., Shinagawa Y., Matsuura K., Hasegawa S., Nakayama T., Fujita H., Watanabe M. (2003). Phylogenetic analysis of spotted fever group rickettsiae based on *gltA*, *17-kDa*, and *rOmpA* genes amplified by nested PCR from ticks in Japan. Microbiol. Immunol..

[55] Birtles R.J., Raoult D. (1996). Comparison of partial citrate synthase gene (gltA) sequences for phylogenetic analysis of *Bartonella* species. Int. J. Syst. Bacteriol..

[56] Billeter S.A., Gundi V.A., Rood M.P., Kosoy M.Y. (2011). Molecular detection and identification of *Bartonella* species in *Xenopsylla cheopis* fleas (Siphonaptera: Pulicidae) collected from *Rattus norvegicus* rats in Los Angeles, California. Appl. Environ. Microbiol..

[57] Sofer S., Gutiérrez R., Morick D., Mumcuoglu K.Y., Harrus S. (2015). Molecular detection of zoonotic bartonellae (*B. henselae*, *B. elizabethae* and *B. rochalimae*) in fleas collected from dogs in Israel. Med. Vet. Entomol..

[58] Casati S., Sager H., Gern L., Piffaretti J.C. (2006). Presence of potentially pathogenic *Babesia* sp. for human in *Ixodes ricinus* in Switzerland. Ann. Agric. Environ. Med..

[59] Springer A., Höltershinken M., Lienhart F., Ermel S., Rehage J., Hülskötter K., Lehmbecker A., Wohlsein P., Barutzki D., Gietl C., Baumgärtner W., Hoedemaker M., Strube C. (2020). Emergence and epidemiology of bovine babesiosis due to *Babesia divergens* on a northern German beef production farm. Front. Vet. Sci..

[60] R Core Team (2022). R: A Language and Environment for Statistical Computing, R Foundation for Statistical Computing, Vienna, Austria. http://www.R-project.org/.

[61] Robert-Koch-Institut (2023). FSME-Risikogebiete in Deutschland (Stand: Januar 2023). Epidemiol. Bull..

[62] Dobler G., Hufert F., Pfeffer M., Essbauer S., Mehlhorn H. (2011). Progress in Parasitology.

[63] Aguero-Rosenfeld M.E., Wang G., Schwartz I., Wormser G.P. (2005). Diagnosis of Lyme borreliosis. Clin. Microbiol. Rev..

[64] Lee S.H., Vigliotti V.S., Vigliotti J.S., Jones W., Williams J., Walshon J. (2010). Early Lyme disease with spirochetemia - diagnosed by DNA sequencing. BMC. Res. Notes.

[65] Anderson J.F., Johnson R.C., Magnarelli L.A. (1987). Seasonal prevalence of *Borrelia burgdorferi* in natural populations of white-footed mice, *Peromyscus leucopus*. J. Clin. Microbiol..

[66] Majerová K., Hönig V., Houda M., Papežík P., Fonville M., Sprong H., Rudenko N., Golovchenko M., Černá Bolfíková B., Hulva P., Růžek D., Hofmannová L., Votýpka J., Modrý D. (2020). Hedgehogs, squirrels, and blackbirds as sentinel hosts for active surveillance of *Borrelia miyamotoi* and *Borrelia burgdorferi* complex in urban and rural environments. Microorganisms.

[67] Skuballa J., Petney T., Pfäffle M., Oehme R., Hartelt K., Fingerle V., Kimmig P., Taraschewski H. (2012). Occurrence of different *Borrelia burgdorferi* sensu lato genospecies including *B. afzelii, B. bavariensis*, and *B. spielmanii* in hedgehogs (*Erinaceus* spp.) in Europe. Ticks Tick Borne Dis..

[68] van Duijvendijk G., Krijger I., van Schaijk M., Fonville M., Gort G., Sprong H., Takken W. (2022). Seasonal dynamics of tick burden and associated *Borrelia burgdorferi* s.l. and *Borrelia miyamotoi* infections in rodents in a Dutch forest ecosystem. Exp. Appl. Acarol..

[69] Khanakah G., Kocianová E., Vyrosteková V., Řeháček J., Kundi M., Stanek G. (2006). Seasonal variations in detecting *Borrelia burgdorferi* sensu lato in rodents from north eastern Austria. Wien. Klin. Wochenschr..

[70] Lesiczka P.M., Hrazdilová K., Majerová K., Fonville M., Sprong H., Hönig V., Hofmannová L., Papežík P., Růžek D., Zurek L., Votýpka J., Modrý D. (2021). The role of peridomestic animals in the eco-epidemiology of *Anaplasma phagocytophilum*. Microb. Ecol..

[71] Gray J.S., Kahl O., Lane R.S., Levin M.L., Tsao J.I. (2016). Diapause in ticks of the medically important *Ixodes ricinus* species complex. Ticks Tick Borne Dis..

[72] Rar V., Tkachev S., Tikunova N. (2021). Genetic diversity of *Anaplasma* bacteria: twenty years later. Infect. Genet. Evol..

[73] Huhn C., Winter C., Wolfsperger T., Wuppenhorst N., Strasek Smrdel K., Skuballa J. (2014). Analysis of the population structure of *Anaplasma phagocytophilum* using multilocus sequence typing. PLoS One.

[74] Jaarsma R.I., Sprong H., Takumi K., Kazimirova M., Silaghi C., Mysterud A., Rudolf I., Beck R., Földvári G., Tomassone L., Groenevelt M., Everts R.R., Rijks J.M., Ecke F., Hörnfeldt B., Modrý D., Majerová K., Votýpka J., Estrada-Peña A. (2019). *Anaplasma phagocytophilum* evolves in geographical and biotic niches of vertebrates and ticks. Parasit. Vectors.

[75] Blazejak K., Janecek E., Strube C. (2017). A 10-year surveillance of Rickettsiales (*Rickettsia* spp. and *Anaplasma phagocytophilum*) in the city of Hanover, Germany, reveals *Rickettsia* spp. as emerging pathogens in ticks. Parasit. Vectors.

[76] Glass A., Springer A., Strube C. (2022). A 15-year monitoring of Rickettsiales (*Anaplasma phagocytophilum* and *Rickettsia* spp.) in questing ticks in the city of Hanover, Germany. Ticks Tick Borne Dis..

[77] Knoll S., Springer A., Hauck D., Schunack B., Pachnicke S., Strube C. (2021). Regional, seasonal, biennial and landscape-associated distribution of *Anaplasma phagocytophilum* and *Rickettsia* spp. infections in *Ixodes* ticks in northern Germany and implications for risk assessment at larger spatial scales. Ticks Tick Borne Dis..

[78] Schex S., Dobler G., Riehm J., Müller J., Essbauer S. (2010). *Rickettsia* spp. in wild small mammals in lower Bavaria, south-eastern Germany. Vector Borne Zoonot. Dis..

[79] Obiegala A., Oltersdorf C., Silaghi C., Kiefer D., Kiefer M., Woll D. (2016). *Rickettsia* spp. in small mammals and their parasitizing ectoparasites from Saxony, Germany. Vet. Parasitol. Reg. Stud. Reports.

[80] Biernat B., Stańczak J., Michalik J., Sikora B., Wierzbicka A. (2016). Prevalence of infection with *Rickettsia helvetica* in *Ixodes ricinus* ticks feeding on non-rickettsiemic rodent hosts in sylvatic habitats of west-central Poland. Ticks Tick Borne Dis..

[81] Hornok S., Kováts D., Csörgő T., Meli M.L., Gönczi E., Hadnagy Z., Takács N., Farkas R., Hofmann-Lehmann R. (2014). Birds as potential reservoirs of tick-borne pathogens: first evidence of bacteraemia with *Rickettsia helvetica*. Parasit. Vectors.

[82] Burgdorfer W., Brinton L.P. (1975). Mechanisms of transovarial infection of spotted fever rickettsiae in ticks. Ann. N. Y. Acad. Sci..

[83] Gilles J., Silaghi C., Just F.T., Pradel I., Pfister K. (2014). Polymerase chain reaction detection of *Rickettsia felis*-like organism in *Archaeopsylla erinacei* (Siphonaptera: Pulicidae) from Bavaria, Germany. J. Med. Entomol..

[84] Gilles J., Just F.T., Silaghi C., Pradel I., Passos L.M., Lengauer H., Hellmann K., Pfister K. (2008). *Rickettsia felis* in fleas, Germany. Emerg. Infect. Dis..

[85] Heglasová I., Víchová B., Kraljik J., Mošanský L., Miklisová D., Stanko M. (2018). Molecular evidence and diversity of the spotted-fever group *Rickettsia* spp. in small mammals from natural, suburban and urban areas of Eastern Slovakia. Ticks Tick Borne Dis..

[86] Silaghi C., Woll D., Mahling M., Pfister K., Pfeffer M. (2012). *Candidatus* Neoehrlichia mikurensis in rodents in an area with sympatric existence of the hard ticks *Ixodes ricinus* and *Dermacentor reticulatus*, Germany. Parasit. Vectors.

[87] Svitálková Z.H., Haruštiaková D., Mahríková L., Mojšová M., Berthová L., Slovák M., Kocianová E., Vayssier-Taussat M., Kazimírová M. (2016). *Candidatus* Neoehrlichia mikurensis in ticks and rodents from urban and natural habitats of South-Western Slovakia. Parasit. Vectors.

[88] Binetruy F., Bailly X., Chevillon C., Martin O.Y., Bernasconi M.V., Duron O. (2019). Phylogenetics of the *Spiroplasma ixodetis* endosymbiont reveals past transfers between ticks and other arthropods. Ticks Tick Borne Dis..

[89] Ogata S., Umemiya-Shirafuji R., Kusakisako K., Kakisaka K., Chatanga E., Hayashi N., Taya Y., Ohari Y., Pandey G.S., Abdelbaset A.E., Qiu Y., Matsuno K., Nonaka N., Nakao R. (2023). Investigation of vertical and horizontal transmission of *Spiroplasma* in ticks under laboratory conditions. Sci. Rep..

[90] Goethert H.K. (2021). What *Babesia microti* is now. Pathogens.

[91] Turner C. (1986). Seasonal and age distributions of *Babesia*, *Hepatozoon*, *Trypanosoma* and *Grahamella* species in *Clethrionomys glareolus* and *Apodemus sylvaticus* populations. Parasitology.

[92] Bown K.J., Lambin X., Telford G., Heyder-Bruckner D., Ogden N.H., Birtles R.J. (2011). The common shrew (*Sorex araneus*): a neglected host of tick-borne infections?. Vector Borne Zoonot. Dis..

[93] Šebek Z., Sixl W., Stünzner D., Valová M., Hubalék Z., Troger H. (1980). Zur Kenntnis der Blutparasiten wildlebender Kleinsäuger in der Steiermark and im Burgenland. Folia Parasitol..

[94] Gray A., Capewell P., Loney C., Katzer F., Shiels B., Weir W. (2019). Sheep as host species for zoonotic *Babesia venatorum*, United Kingdom. Emerg. Infect. Dis..

[95] Cutler S.J., Vayssier-Taussat M., Estrada-Peña A., Potkonjak A., Mihalca A.D., Zeller H. (2021). Tick-borne diseases and co-infection: current considerations. Ticks Tick Borne Dis..

